# CRISPR/Cas9 ribonucleoprotein mediated DNA-free genome editing in larch

**DOI:** 10.48130/forres-0024-0033

**Published:** 2024-10-31

**Authors:** Miaomiao Ma, Chan Zhang, Lijing Yu, Jingli Yang, Chenghao Li

**Affiliations:** State Key Laboratory of Tree Genetics and Breeding, Northeast Forestry University, Harbin 150040, China

**Keywords:** Transgene-free genome editing, Cas9-gRNA RNPs, *Larix kaempferi*, Particle bombardment, Somatic embryogenesis

## Abstract

Here, a DNA-free genetic editing approach is presented for larch by delivering ribonucleoprotein complexes (RNPs) of CRISPR/Cas9 through particle bombardment. The detailed procedure encompasses creating a transgenic system *via* particle bombardment for the transformation of embryogenic callus, validating the functionality of RNPs, optimizing coating and delivery techniques, enhancing somatic embryo maturation, regenerating plantlets, and precisely identifying mutants. The optimal particle bombardment parameters were determined at 1,100 psi and a distance of 9 cm and the editing efficiency of the targets was verified *in vitro*. Subsequently, the RNPs were transferred into the embryogenic callus. Mutant plants were obtained in targets 1 and target 2. The efficiencies of obtaining albino somatic embryos were 1.423% and 2.136%, respectively. A DNA-free particle bombardment transformation method suitable for larch has been established. The present study demonstrates that the DNA-free editing technology has been successfully implemented in larch. This method can achieve targeted genome editing in the larch genome, avoiding the risks of genomic integration and the lengthy breeding cycles associated with traditional transgenic methods. Moreover, it may be widely applicable for producing genome-edited conifer plants and holds great promise for commercialization.

## Introduction

In recent years, the CRISPR/Cas9 system has been widely employed in plants for introducing genome modifications and is paving the way for the precise improvement of crop traits^[1]^. Typically, CRISPR/Cas9 DNA constructs are delivered into plant cells through *Agrobacterium tumefaciens*-mediated T-DNA transfer or biolistic bombardment. Once inside, they are expressed, cleave target sites, and produce mutations^[2]^. During this process, there is a high likelihood that the CRISPR/Cas9 constructs are integrated into the plant genome^[3]^. This increases the risk of unwanted genetic changes, with transgene integration and off-target mutation being the most significant concerns. Moreover, once within the recipient cells, the CRISPR/Cas9 sequence may be degraded, and the resulting fragments can act as filler DNA in the double-stranded break repair process and be inserted into intended and/or unintended genomic sites^[4]^. This can contaminate the genome and introduce foreign DNA. As a result, currently, the biosecurity of genome-edited plants are a major public concern^[5]^. In response to this concern, significant efforts are being made to optimize CRISPR/Cas9-mediated genome editing to avoid transgene integration and off-target mutations. Subsequently, Woo et al.^[6]^ transfected preassembled complexes of purified Cas9 protein and guide RNA into plants and demonstrated that the use of preassembled CRISPR/Cas9 ribonucleoproteins (RNPs) completely avoids transgene integration and greatly reduced off-target mutations. The direct delivery of Cas9-sgRNA RNP complexes induce mutations at target sites immediately after delivery and decomposes rapidly by endogenous proteases, reducing off-target mutations without compromising on-target efficiency. Thus, RGEN RNPs are regarded as a groundbreaking technology for producing DNA-free genetically edited crop plants^[7]^.

Larch (*Larix spp.*), being one of the most abundant conifer trees in the northern hemisphere, holds significant ecological and economic value^[8]^. In recent years, gene editing of coniferous trees has emerged as a prominent topic in biological science and forestry. Conventionally, CRISPR/Cas9 constructs are introduced into cells through *Agrobacterium*-mediated T-DNA transfer, which poses a risk of genomic integration of these constructs. Given the long breeding cycles of trees, strategies such as genetic segregation used to achieve DNA-free editing in annual crops are inefficient for forestry applications^[9]^. Therefore, the adaptation of non-transgenic, efficient genome editing methods is essential. The cutting-edge CRISPR/Cas9 RNPs technology, administering Cas9-sgRNA complexes directly to cells offers a robust solution for DNA-free genetic editing in plants^[10]^. We could develop a CRISPR/Cas9 RNP-mediated genome editing method for efficient and specific genome editing of major conifers, using *L. kaempferi* as experimental material and targeting the phytoene desaturase (*PDS*) gene. The *PDS* gene has been widely used as a phenotypic marker to rapidly standardize the establishment of CRISPR/Cas9 in a new plant system. Since its disruption leads to a color phenotype change, it makes it easy to screen mutant lines^[11]^. Accordingly, the present study aims to investigate the effectiveness of genome editing by directly delivering purified CRISPR-Cas9 RNPs to the embryogenic callus of *L. kaempferi*. We can also provide a reference for cultivating mutant coniferous tree materials with excellent commercial prospects.

## Materials and methods

### Embryogenic callus culture medium

Embryogenic calluses of *L. kaempefri* were induced from immature zygotic embryos extracted from seeds collected between June 30^th^ and July 7^th^, 2021^[12]^. The methods for inducing embryogenic callus are elaborated in our previous study^[13]^.

The embryogenic calluses were initially cultured on a proliferation medium (BM_1_), which is comprised of BM^[14]^ basal medium supplemented with 0.2 μM 6-BA, 0.4 μM 2,4-D, 0.1 g/L inositol, 0.5 g/L casein hydrolysate, 1.125 g/L L-glutamine, 30 g/L sucrose, and 7% (w/v) agar. This culture was maintained in darkness at 22 ± 1 °C. Before transitioning to embryonic callus maturation, the embryogenic calluses were cultured on a plant growth regulator-free transition medium, BM_2_, for one week. The maturation medium, BM_3_, comprises 30 mg/L abscisic acid (ABA), 1.0 g/L inositol, 1.0 g/L casein hydrolysate, 2.25 g/L L-glutamine, and 9% (w/v) sucrose. For the germination of mature somatic embryos, a half-strength MS medium^[15]^ was employed. All culture media were adjusted to pH 5.8 before being autoclaved at 121 °C for 20 min Sterilization was carried out by autoclaving. Details of the culture media compositions can be found in Supplementary Table S1.

### Determination of particle bombardment-mediated parameters

#### Preparation of embryogenic callus

One gram of healthy and vigorous embryonic calluses was selected and placed at the center of a petri dish with a diameter of 2 cm. The petri dish was pre-cultured in darkness for two days before being subjected to particle bombardment and was filled with a 0.5 cm layer of BM_1_ culture medium.

#### Plasmid extraction

The plant transformation vector pBI121 utilized in this study contains a selective marker gene Hygromycin B and a reporter gene encoding β-glucuronidase (*GUS*), both of which are controlled by a 35S promoter derived from the cauliflower mosaic virus (CaMV) (Supplementary Fig. S1). Plasmid DNA was introduced into *E. coli* DH5α cells. High-quality DNA for particle bombardment transformation was prepared using the gradient equilibrium centrifugation method of the EndoFree Maxi Plasmid Kit (TIANGEN, Beijing, China). Supercoiled plasmid DNA with a concentration of 1 μg/μl, was directly used in the transformation experiments.

#### Gold particle coating with DNA

First, 60 mg of gold powder is weighed. Then, 700 μL of 75% anhydrous ethanol was added and thoroughly vortexed for 10 min. The mixture was left to stand at room temperature for 10 min, followed by centrifugation at 1,500 rpm for 5 min. The supernatant was removed and discarded. Next, 700 μL of sterile water was added, vortexed for 5 min, and centrifuged at 1,500 rpm for 5 min before discarding the supernatant. This washing process was repeated twice. One mL of 50% glycerol was then added to the washed gold particles and mixed well by vortexing to obtain a gold powder with a final concentration of 60 mg/mL. The prepared gold suspension was stored at 4 °C.

Fifty μL of the 60 mg/mL gold suspension was then transferred into a 1.5 mL Eppendorf tube and left to settle for 1 min to ensure complete suspension. Next, 5 μg of plasmid DNA and 50 μL of CaCl_2_ (2.5 M) were sequentially added to the gold suspension, and gently vortexed for a few seconds. Twenty μL of spermidine (0.1 M) was added and vortexed for 2 min, then incubated on ice for 2 min. Vortexing was repeated for an additional 2 min followed by 2 min of ice incubation. Subsequently, the mixture was centrifuged for 45 s at 10,000 rpm and the supernatant discarded. The pellet was then washed with 500 μL of anhydrous ethanol, vortexed for 2 min, and centrifuged for 1 min at 10,000 rpm. These steps were repeated twice. Finally, the pellet was suspended in 60 μL of anhydrous ethanol. These volumes are designed for six bombardments.

#### Procedure for particle bombardment

In this study, the Biorad PDS-1000/He desktop particle bombardment system was employed. Before bombarding pBI121-*GUS*, the relevant components were thoroughly sterlized with 75% ethanol, including the external and internal parts of the particle bombardment equipment, the macro carrier launch assembly, the rupture disc retaining cap, and the target disk holder. Ten μL of the DNA-gold mixture suspension was applied to the center of the micro-carrier and left to air dry for a few minutes before initiating the bombardment procedure. The particle bombardment parameters consisted of different combinations of rupture disc pressures (900, 1,100, and 1,350 psi) and target distances (9, 12, and 15 cm from the rupture discs to the target callus). The pre-cultured callus tissue was bombarded according to these parameters. The details of the particle bombardment technique were elaborated by Wang et al.^[16]^. For each unique parameter combination, there were six bombardments, and each session targeted one plate of callus.

#### Evaluation of parameters influencing transformation

After a two-day dark incubation following particle bombardment, the embryogenic callus in each petri dish were divided into 20 small pieces and cultured in BM_1_ for 5 d. Subsequently, the callus was transferred to the selection medium BM_4_, which contained 3 mg/L hygromycin. The total number of hygromycin-resistant callus pieces was counted under each parameter after 21 d, and each resistant piece was considered a putative transgenic line. Three callus tissue discs were used under each parameter to assess the stable transformation effect following particle bombardment.

For *GUS* staining, 5-bromo-4-chloro-3-indolyl glucuronide-β-glucuronidase (X-gluc) was used according to the method described by Li et al.^[17]^. The stained samples were then incubated at 37 °C in darkness for 12 h and subsequently examined under a light microscope. Statistical analysis involved quantifying the number of *GUS*-stained spots under various experimental conditions. Each parameter was tested in three independent replicates to evaluate the immediate conversion results after particle bombardment.

#### Molecular analysis of transformants

Genomic DNA was extracted from 100 mg of both transformed and wild type (WT) embryogenic callus samples by using the cetyltrimethylammonium bromide (CTAB) method^[18]^. Thirteen clumps were then subjected to PCR screening to detect the presence of the *GUS* gene in transgenic embryogenic callus. For RNA extraction, embryogenic callus samples from the transgenic and WT groups were processed using the FastPure® Plant Total RNA Isolation Kit (Bioteke, Beijing, China). The extracted RNA was subsequently reverse-transcribed into cDNA using the HiScript® III 1st Strand cDNA Synthesis Kit (Vazyme, Nanjing, China).

After cDNA synthesis, quantitative real-time PCR (qRT-PCR) reactions were performed in a 20 μL reaction volume containing 2×TransStar® Top Green qPCR Super Mix (Transgen, Beijing, China), 10 ng of cDNA, and primers at a concentration of 10 mM. The glyceraldehyde 3-phosphate dehydrogenase (GAPDH) gene was used as an internal control gene^[19]^. The primer sequences used are detailed in Supplementary Table S2.

#### Plant regeneration of transgenic somatic embryos

**Figure 1:**
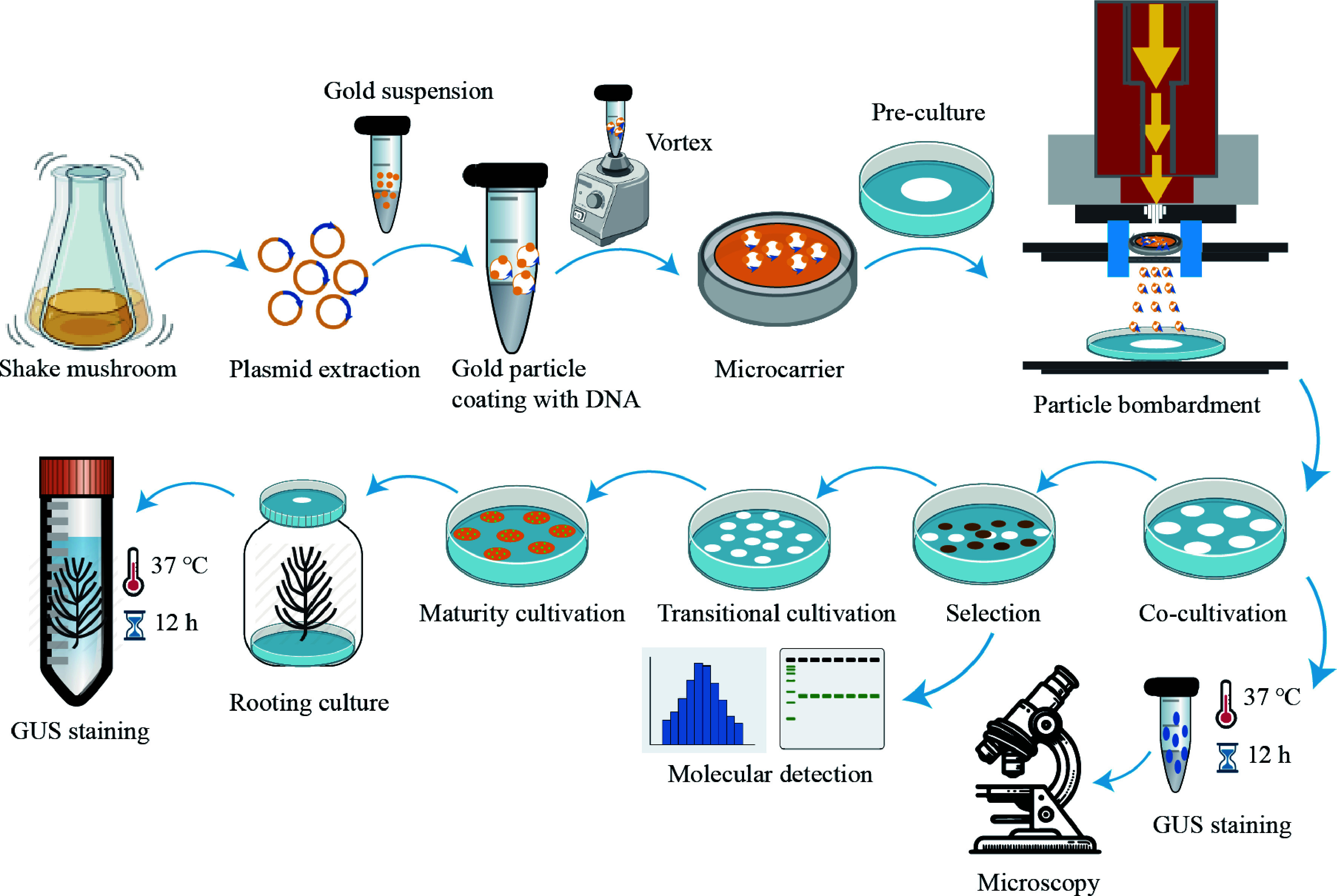
The flowchart for determining optimal particle bombardment parameters.

The hygromycin-resistant embryogenic callus was subsequently transferred to a PGR-free BM_2_ medium for one week under dark conditions at 22 ± 1 °C. After that, the embryogenic callus was transferred to the maturation medium BM_3_ for 45 to 60 d to induce somatic embryos. The resulting mature somatic embryos were then transferred to the 1/2 MS germination medium to initiate germination and rooting. GUS staining was carried out on the resultant somatic embryo plants. The entire process diagram is shown in Fig. 1.

### *In vitro* validation of the targets of *LkPDS*

#### Selection of target sequences and vector construction

The complete cDNA sequence of the *LkPDS* (NCBI accession number BSBM01000076.1) gene from the *L. kaempferi* genome was cloned by amplifying the gene with specific primers (detailed in Supplementary Table S3) and using 2,000 ng of *L. kaempferi* genomic cDNA. The PCR reaction mixture included 100 μL of 10 × KOD buffer, 40 μL of dNTP (8 mM), 10 μL each of PDS-F (10 μM) and PDS-R (10 μM) primers, 8 μL of KOD FX (TOYOBO-KFX-101) polymerase, and 32 μL of nuclease-free water, for a total volume of 200 μL. PCR amplification was carried out under the following conditions: initial denaturation at 94 °C for 2 min, followed by 30 cycles of 98 °C for 10 s, 60 °C for 30 s, 68 °C for 1 min and 30 s, and a final extension at 68 °C for 5 min.

The CRISPR-P 2.0 online tool was used to automatically identify and design the target sequences of the *LkPDS* gene^[20]^, and the RNA fold web tools^[21]^ were used to ensure their efficacy and specificity in editing the *LkPDS* gene. To target the *LkPDS* gene effectively, five distinct targets were selected within the first exon (Supplementary Fig. S2). The genomic location of the *LkPDS* gene was predicted based on the genome sequence of *L. kaempferi*. For further details on the NCBI link to the *L. kaempferi* genome containing the *LkPDS* gene, please visit *L. kaempferi* genome assembly LKA_r1.0 on the NCBI - NLM website. The specific sequences of primers are provided in Supplementary Table S4 for reference. The GC content was analyzed using an online tool (https://crm.vazyme.com/cetool/tmcal.html), while the efficacy of the sgRNA was predicted through the CRISPR Efficiency Predictor (www.flyrnai.org/evaluateCrispr)^[22]^.

The recombinant vector was assembled by using the fragment sequence and the pAbAi vector (Supplementary Fig. S3) as the target DNA fragments for *in vitro* validation. Both the fragment sequence and the pAbAi vector contained cleavage sites compatible with *Sac I* and *Sal I* enzymes. To merge the target fragment with the vector, 450 ng of the fragment sequence insert, 150 ng of the pAbAi vector, 2 μL of 5 × T4 DNA ligase buffer, 1 μL of T4 DNA ligase, and 2 μL of nuclease-free water were combined in a total volume of 10 μL. The ligation reaction was conducted at 25 °C for 10 min.

#### Cas9 protein puriﬁcation

The pMJ915 vector (Supplementary Fig. S4), which contains the Cas9 coding sequence flanked by a pair of T7 promoters and an MBP sequence was utilized^[23]^ to express Cas9 protein. The expression of Cas9 protein was induced in the *E*. *coli* Rosetta strain at 21 °C for 14 h. Subsequently, the protein was purified by nickel affinity chromatography and desalted using ice-cold PBS buffer with a molecular weight cut-off (MWCO) filter. The purity and concentration of the Cas9 protein were evaluated using the Bradford protein assay. Then, the concentration of the Cas9 protein was adjusted to 1 μg/μL for subsequent experimental procedures.

#### ***In vitro*** transcription of sgRNA

Transcription templates were prepared through a specific PCR protocol employing specific primers is detailed in Supplementary Table S5. The transcription process was carried out using the NEB T7 Quick High Yield RNA Synthesis Kit in accordance with the provided instructions.

#### ***In vitro*** cleavage of CRISPR/Cas9 RNPs

The recombinant vector outlined in Supplementary Table S6 was linearized utilizing the *BstB I* enzyme. The target DNA fragments containing the designated target site were purified and eluted with RNase-free water. A combination of Cas9 protein (2 μg), gRNA (2 μg), and the purified 12 μL linearized target DNA (250 ng) was prepared in a reaction buffer consisting of 0.8 μL 1 M phosphate buffer (pH 7.5), 2.18 μL PBS, 1 μL 100 mM MgCl_2_, and 0.02 μL 1 M DTT to achieve a total volume of 20 μL. This mixture was digested at 37 °C for 1 h as detailed in Supplementary Table S6. The resultant products were purified using QIAquick spin columns for PCR purification. Subsequently, the purified products were subjected to analysis on a 1% agarose gel, and the cleavage activity was assessed by quantifying the number of digested products relative to the total input target DNA quantity.

### Particle bombardment of Cas9 RNP complexes

For each bombardment, 2 μL each of Cas9 protein and gRNA were mixed in a reaction buffer containing 20 mM HEPES (pH 7.5), 150 mM KCl, 10 mM MgCl_2_, and 0.5 mM DTT, totaling 10 μL. This mixture was then incubated at 25 °C for 10 min. Subsequently, 5 μL of 0.6 mM gold nanoparticles were added. The particles coated with this mixture were evenly distributed onto the carrier and allowed to air-dry at room temperature for approximately 10 min^[24]^. The bombardment systems established parameters, including a helium pressure of 1,100 psi and a bombardment distance of 9 cm, were utilized for the effective delivery of the Cas9 RNPs into the embryogenic callus.

### Mutation identiﬁcation

The embryogenic callus that had been treated with Cas9 RNPs through biolistic bombardment was dissected into small fragments, each with a diameter of approximately 0.5 cm, and cultured on BM_2_ medium. After another week, the fragments were transferred to BM_3_ medium to promote the development of somatic embryos. During a maturation period of 45−60 d, the somatic embryos were transferred to 1/2 MS germination medium to initiate germination and plant regeneration. Initial mutation screening was carried out by examining variations in the color of the germinating somatic embryos. Mutations suspected to be positive were precisely identified using specific primers and confirmed by Sanger sequencing, with reference to the primers detailed in Supplementary Table S7. A detailed statistical analysis was performed on the mutant somatic embryos derived from various targets, including data such as the total number of somatic embryos obtained from six grams of embryonic callus and the count of albino somatic embryos. The editing efficiency was evaluated by determining the percentage of albino embryos relative to the total number of embryos produced.

### Statistical analysis

Data analysis was conducted using a least significance difference (LSD) in one-way analysis of variance (ANOVA) to determine statistical significance, which was set at a threshold of *p* ≤ 0.05. The varying levels of significance, indicated by labels from a to g, represent a gradient of significance from highest to lowest.

## Results

### Determination of optimal parameters for particle bombardment

**Figure 2:**
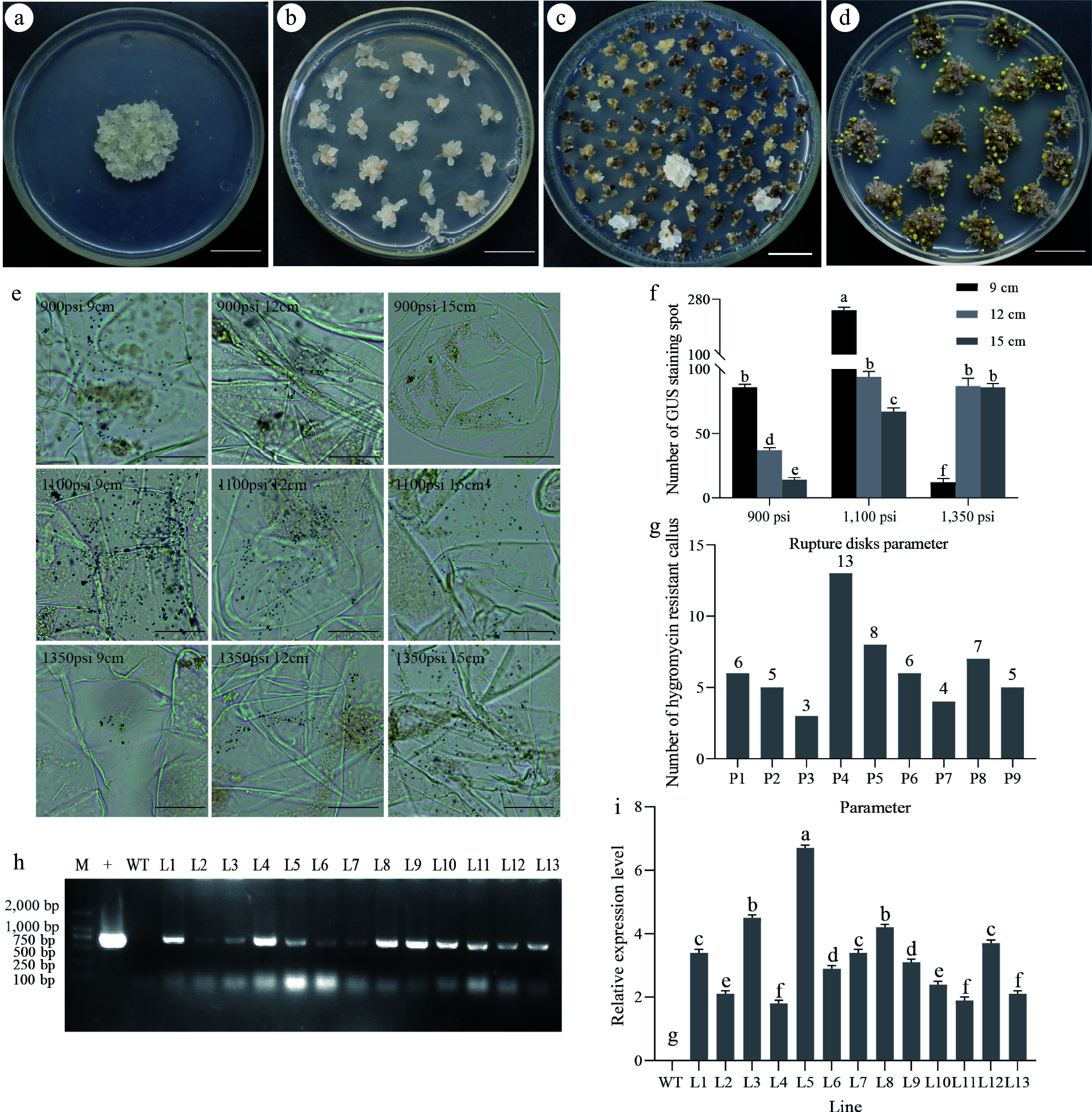
Transformation experiment results *via* particle bombardment. (a) Pre-culture before particle bombardment; (b) Subculture after particle bombardment; (c) Hygromycin-resistant embryogenic callus shown visually; (d) Induction of somatic embryos, (a)−(d) bar =1.5 cm. (e) Histochemical staining for *GUS* expression after particle bombardment under various parameters in embryogenic callus, bar = 50 μm; (f) The statistical analysis of GUS blue spot numbers under various particle bombardment parameters. Mean ± standard deviation, n = 3 (ANOVA; *p* ≤ 0.05); (g) The number of hygromycin-resistant callus under different parameters (The total sum of all hygromycin-resistant callus tissues in six replicates under each parameter. P1: 900 psi and 9 cm, P2: 900 psi and 12 cm, P3: 900 psi and 15 cm, P4: 1100 psi and 9 cm, P5: 1,100 psi and 12 cm, P6: 1,100 psi and 15 cm, P7: 1,350 psi and 9 cm, P8: 1,350 psi and 12 cm, P9: 1,350 psi and 15 cm). (h) Polymerase chain reaction (PCR) analysis of the ß-glucuronidase (*GUS*) gene (700 bp) at DNA levels in transgenic lines subjected to the parameters 1,100 psi and 9 cm; (i) Quantitative real-time (qRT)-PCR quantification of *GUS* gene expression levels under the parameters 1,100 psi and 9 cm, with wild-type (WT) as the negative control. Lines 1−13 denote transgenic lines of embryogenic callus. Data are represented as the mean from a minimum of three replicates. Different letters (a−g) above the column chart indicate statistically significant differences determined by an ANOVA test. Mean ± SD, n = 3. (ANOVA test; *p* ≤ 0.05).

To ensure the conversion efficiency of particle bombardment transformation, the genetic transformation of embryogenic callus were initiated using the PBI121-*GUS* vector particle bombardment under various settings with different combinations of rupture disk pressure and target distance parameters. The pre-culture of the embryogenic callus before bombardment is shown in Fig. 2a. After bombardment, the embryogenic callus was cultured for a week (Fig. 2b) before being transferred to hygromycin select. The hygromycin-resistant callus is shown in Fig. 2c. The induction results of somatic embryos are shown in Fig. 2d.

The microscopic examination results of *GUS* histochemical staining of calluses after bombardment in each parameter (Fig. 2e), along with the statistics of *GUS* blue spots (Fig. 2f), indicated that the optimal bombardment conditions were identified as 1,100 psi pressure and 9 cm distance. The total number of callus tissue clumps for resistance selection is 720 under each parameter. At 900 psi, the number of hygromycin-resistant callus is 6, 5, and 3 respectively. At 1,100 psi, the number of hygromycin-resistant callus is 13, 8, and 6 respectively. And at 1,350 psi, the number of hygromycin-resistant callus is 4, 7, and 5 respectively (Fig. 2g). The evaluation of transformation efficiency under diverse parameters showed that the transformation efficiency was most significant at 1,100 psi and a 9 cm distance (P4: 13). Furthermore, molecular assessments involving PCR of hygromycin-resistant embryogenic callus lines under these optimized conditions (Fig. 2h) and quantitative real-time PCR for *GUS* gene expression (Fig. 2i) affirmed successful gene integration and expression within the *L. kaempferi* genome.

**Figure 3:**
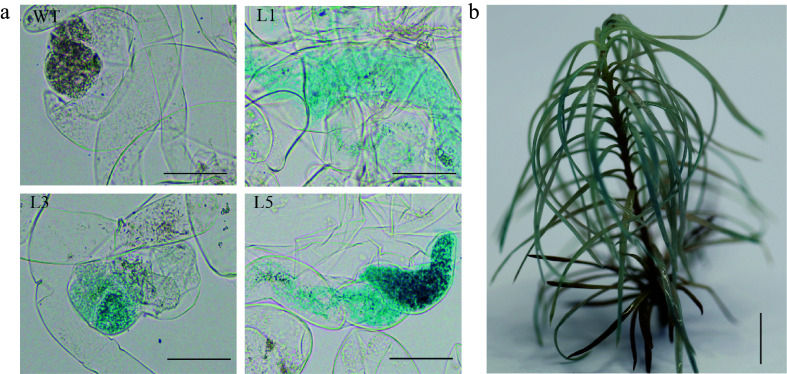
Histochemical staining for ß-glucuronidase (GUS) activity in the transgenic embryogenic callus and the transgenic plant. (a) Histochemical staining for GUS activity in WT and transgenic embryogenic callus (L1, L3 and L5 represent different lines), Scale bar = 100 μm; (b) Histochemical staining for GUS activity in regenerated plants, Scale bar = 2 cm.

Moreover, the *GUS* activity evidenced by histochemical staining in both transgenic callus (Fig. 3a) and corresponding transgenic plants (Fig. 3b) confirmed the stable hereditary transmission of the *GUS* gene from embryonic callus to plant regeneration.

### *In vitro* validation of the targets

**Figure 4:**
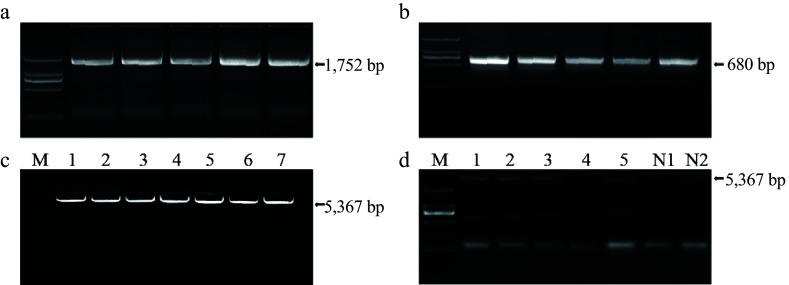
*In vitro* validation process for five target sites. (a) Cloning of the *LkPDS* gene. M, DL2000 marker; (b) Detection of the recombinant vector. M, DL2000 marker; (c) Linearized recombinant vector. M, DL15000 marker; (d) *In vitro* validation results. 1−5: represent five specific targets; Negative control: N1: gRNA(-), N2: Cas9(-); M, DL2000 marker.

To evaluate the editing efficacy of the *LkPDS* gene targets *in vitro*, the full-length cDNAs encoding the *LkPDS* gene were cloned. The *LkPDS* gene is 1,752 bp in length (Fig. 4a), and the editing sites were precisely identified using CRISPR-P v.2.0 software. According to the selection criteria for efficient sgRNAs and genomic location prediction of the *LkPDS* gene (Supplementary Fig. S2), five targets on the first exon were selected (Table 1).

**Table 1:** Sequences of the five target sites, GC content and their corresponding predicted sgRNA efficiency.

Name	Sequence (5'-3')	GC (%) predicted	sgRNA efficiency
Target 1	GCAGCAGTCTGTCATCTGCG	60	4.7625
Target 2	TGCGCTCTGTGAAAAAGAAA	40	5.03752
Target 3	AAAGGGATCGAAACGCGACG	55	4.72023
Target 4	AGGTTTGGCTGGCTTGTCAA	50	6.36878
Target 5	GAGGCAAGAGATGTTCTTGG	50	7.32143

A vector containing the target sites was constructed for *in vitro* validation. The recombinant vector result confirmed by the vector primer and gene primer is 680 bp (Fig. 4b). The length of the linearized recombinant vector is a combination of gene length and vector length, which is 5,367 bp (Fig. 4c). The sgRNA was transcribed *in vitro* and mixed with purified Cas9 protein. The resulting RNPs exhibited effective cleavage activity *in vitro* is shown in Fig. 4d. In Fig. 4d, lanes 1−5 represent the cleavage results of the five targets, respectively. The two electrophoretic bands in lanes 1−5 are the result of the recombinant vector breaking at the target location. Lanes N1 and N2 are negative controls for the absence of Cas9 and sgRNAs in the reaction, respectively. This result indicates that the RNPs can function at the target location of genes, and the process requires the combined action of cas9 protein and sgRNA.

### The gain and identiﬁcation of mutants

**Figure 5:**
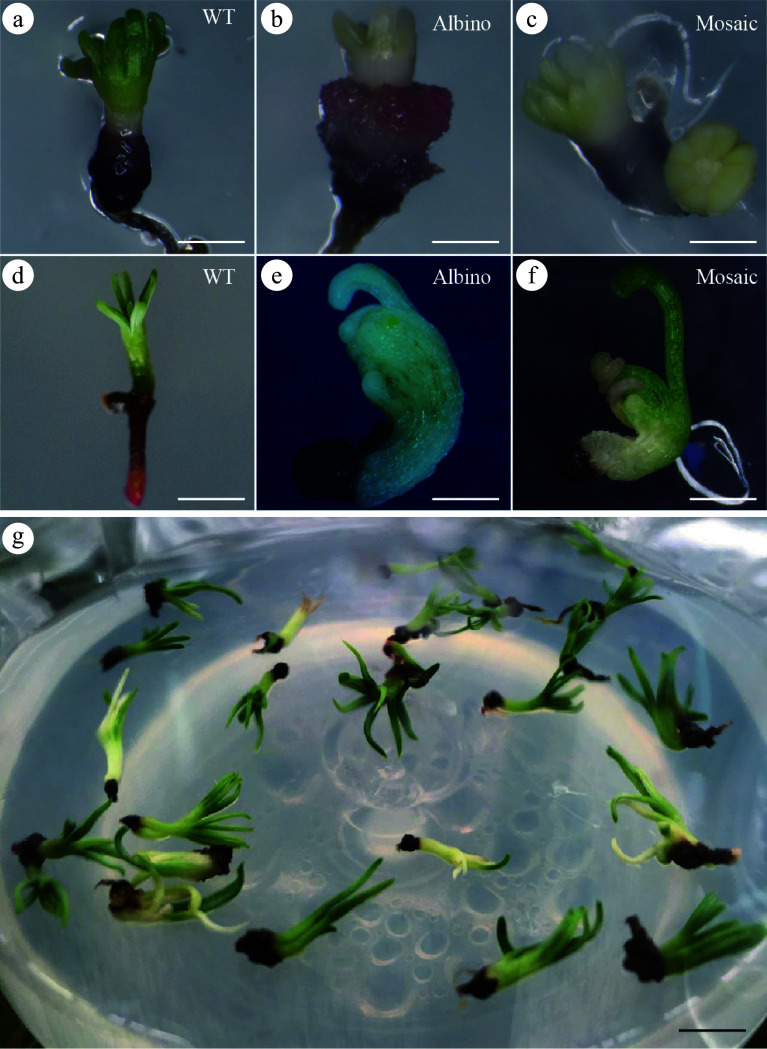
Albino and mosaic mature somatic embryos developed from embryogenic callus bombarded with Cas9/gRNA particles. Mature somatic embryos exposed to light for 4 weeks; (a) WT, bar = 1.5 mm; (b) albino, bar = 1 mm; (c) mosaic, bar = 1 mm. Albino and mosaic mutant plants after particle bombardment; (d) Somatic embryo plant of WT, bar =1 cm; (e) Somatic embryo of albino, bar =2 mm; (f) Somatic embryo plant of mosaic, bar = 2 mm; (g) Depicts somatic embryo plants after 8 weeks of light exposure, bar = 1 cm.

Proteins and sgRNA were synthesized *in vitro* and then the Cas9 RNPs complex delivered into embryogenic callus employing particle bombardment at settings of 1,100 psi and 9 cm. After a week of transitional culture, the embryonic callus treated with the gene gun was transferred to the somatic embryo maturation medium to facilitate further development. Five months after treatment, somatic embryo-derived plants were successfully obtained. Throughout the entire process, the growth phenotypes of WT, albino, and mosaic plants were meticulously documented at various developmental stages (Fig. 5a−f). In the germination culture phase, WT plants showed robust elongation and developed healthy roots (Fig. 5d). In contrast, albino plants displayed severely stunted growth and ultimately turned black and died (Fig. 5e). Mosaic plants had significantly slower growth rates compared to WT plants (Fig. 5f). Upon eight weeks of light exposure, the phenotypic characteristics of the somatic embryo plants were evident as shown in Fig. 5g.

**Figure 6:**
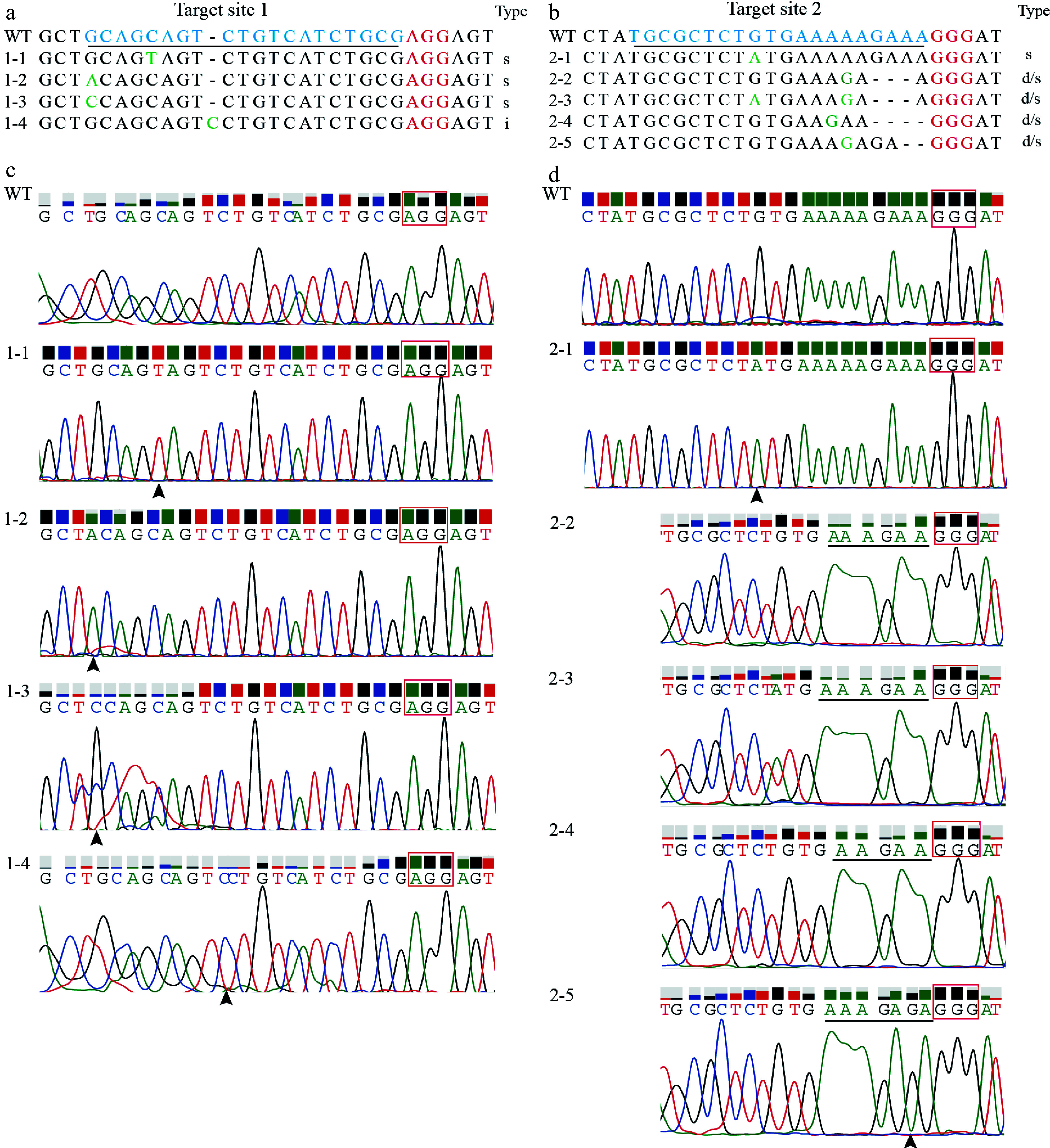
The editing results and data statistics of mutant plants. (a) Displays Sanger sequencing results at the target site in mutated somatic embryo plants. Blue indicates the target site and red denoting PAM sites. Nucleotide insertion, deletion, and substitution are marked as 'i', 'd', and 's', respectively. (b) Sequences at target site 1 in mutant somatic embryo plants. The black arrows indicate the mutation sites. PAM sites are highlighted in red spaces. (c) The results 1-1, 1-2 and 1-3 are single base mutations at the black arrows sites. The sequencing chromatograms of 1-2 and 1-3 are characterized by significantly double peaks. The result 1-4 is a single base insertion at the black arrow site. (d) The result 2-1 is single base mutations at the black arrows sites. The sequencing chromatograms of the 2-2 to 2-5 were characterized the deletion of bases at the black line. The result 2-5 is also showed single base mutations at the black arrows sites.

Subsequent sequencing of these plants using specific primers validated targeted edits in the *LkPDS* gene at targets 1 and 2 (Fig. 6a, b). An analysis of the sequencing peak plots showed that site 1 in the chromatograms predominantly displayed double peaks, signifying heterozygous mutations (Fig. 6c). Target site 2 predominantly exhibited biallelic mutations (Fig. 6d), leading to a higher proportion of albino somatic embryo plants. Simple base mutations appeared to be more frequently associated with the mosaic phenotype. Moreover, the numbers of somatic embryo plants, as well as the counts of albino and mosaic plants at five target sites, were recorded (Table 2).

**Table 2:** The number of albino and mosaic transgenic somatic embryo plants among the five targets.

Target site number	Total number of somatic embryos	Number of albino somatic embryo plants	Number of mosaic somatic embryo plants
Target site 1	281	1	3
Target site 2	234	4	1
Target site 3	316	0	0
Target site 4	162	0	0
Target site 5	244	0	0

## Discussion

Gene editing in coniferous trees, especially larch, is attracting attention in the fields of biological science and forestry. In this study, an effective genetic editing system is presented and, for the first time, the potency of CRISPR/Cas9 RNPs demonstrated as a powerful instrument for genome editing in larch. This advancement is poised to greatly accelerate research into genetic breeding and the development of new germplasm in larch.

The mediation system of RNPs is crucial for the efficiency of larch gene editing. In the present research, the *GUS* reporter gene was employed to determine the optimal parameters for particle bombardment transformation of embryogenic calluses. Various parameter combinations were explored and detailed statistics conducted to identify the most effective transformation settings. Subsequently, robust callus tissue and plants were successfully developed. This comprehensive approach outlines a solid protocol for particle bombardment-mediated genetic transformation in larch. Proteins and sgRNA were synthesized *in vitro* and these optimized parameters applied to transform the CRISPR/Cas9 RNPs into embryogenic calluses. Compared to the *Agrobacterium*-mediated approach, which requires the construction of vector plasmids, the present direct delivery method enables immediate genomic editing with RNPs that are only transiently expressed. Previous studies such as Subburaj et al.^[25]^ have shown that plasmid-based methods are significantly less effective than RNPs. Moreover, compared to the PEG-mediated method, particle bombardment is simpler, more efficient, and applicable to a broader range of materials. This efficient and robust genetic transformation system has enabled us to establish a CRISPR/Cas9-based genome editing tool specifically tailored for larch. Consequently, the proposed method is not only more precise but also acts more rapidly in altering the larch genome. This highlights its potential as a transformative tool in forest genetic research and applications.

Appropriate explants are crucial for successful gene editing in larch^[26]^. Although gene editing of tree species is not yet widespread, the utilization of protoplasts for initial proof-of-concept studies has been relatively common in certain tree species^[27,28]^. Additionally, genome editing with RNPs has been effectively implemented in various tree species utilizing protoplasts^[29,30]^. Compared to the regeneration challenges associated with transformed protoplasts, the embryogenic callus is an advantageous receptor for gene editing due to its widely available source material, rapid reproduction rate, receptiveness to exogenous genes, and high transformation efficiency. Furthermore, the application of RNPs in somatic embryogenic cell cultures has been documented, with remarkable success in achieving CRISPR/Cas9 genome editing in *Pinus radiata*^[31]^. Similar approaches have led to the generation of gene-edited mutants in embryogenic tissues of *Picea glauca*^[32]^, highlighting the suitability of using callus tissue for gene editing in coniferous trees. In the present study, taking advantage of the unique characteristics of larch, embryogenic calluses combined with somatic embryogenesis technology were chosen to obtain the mutants (Fig. 3a−d). Compared to other types of receptor materials, such as leaves, protoplasts, and somatic embryos, the embryogenic calluses demonstrated high genetic stability, which ensured that the edited plants remained consistent with those of the original embryogenic calluses. The stronger proliferative capacity of the embryogenic calluses enables rapid propagation and the production of a greater number of genetically edited plants. The mutations introduced during the editing process were faithfully transmitted from the embryogenic calluses to the regenerated plants.

A detailed analysis of RNPs-mediated mutagenesis uncovered significant differences between the efficiency of *in vitro* cleavage in embryogenic calluses and the overall gene editing efficiency across various target sites (Fig. 4, Table 2). This variation in efficiency has been supported by other studies, including those by Liu et al.^[33]^ and An et al.^[34]^. Notably, the present data indicated exceptionally higher editing efficiency at target site 2 compared to other sites, which can be largely ascribed to its lower GC content. Low GC content has been previously suggested by Tsai et al.^[35]^ to reduce the likelihood of off-target effects, thereby enhancing the precision of the edits. Furthermore, the RNA sequence for the sgRNA at target site 2 was found to contain a significant proportion of Uracil. This composition enhances sgRNA specificity and potentially reduces its degradation or disintegration, as proposed by Pallarès Masmitjà et al.^[36]^. These findings emphasize the crucial role of strategic target site selection in maximizing the efficacy of CRISPR/Cas9 applications for genetic improvement in larch. Additionally, disparities have been observed between the efficiency of *in vitro* DNA cleavage and that achieved through protoplast transfection, a phenomenon that might be associated with the architecture and modifications of chromatin as suggested by Park et al.^[37]^. These structural differences could influence the accessibility of the target DNA sites and the overall efficiency of the gene editing process. Given these insights, it is essential to select sgRNAs that are both customized for specific genetic targets and compatible with the cellular environment to ensure optimal integration and function during in-cell transfection.

Mosaicism is a phenomenon that cannot be overlooked in the results of gene editing. In the present research, it was observed that the mutants frequently displayed mosaicism (Fig. 6g), a phenomenon also reported in genome editing of other coniferous species such as *Pinus radiata*^[31]^, *Picea glauca*^[32]^, and Japanese cedar^[38]^. Mosaicism can occur due to the delayed action of the Cas9 enzyme during the rapid division of embryogenic tissues, as suggested in previous studies^[39,40]^. Additionally, the sustained activity of CRISPR/Cas9 may contribute to this phenomenon^[38]^. Another aspect that might influence the occurrence of chimeric mutations is the culture temperature. For instance, Xiang et al.^[41]^ identified that the optimal activity temperature for the CRISPR/Cas9 system is 37 °C. Deviations from this optimal temperature can lead to uneven activation of the Cas9 enzyme, thereby potentially increasing the likelihood of generating chimeric mutants. These factors-delayed Cas9 cleavages, the ongoing activity of CRISPR/Cas9, and sub-optimal culture temperatures could cause the formation of plants composed of cells that have been edited differently, contributing to the chimeric nature of the mutants. Considering these variables can be crucial when planning and executing gene editing in larch, as they significantly impact the homogeneity and stability of the desired genetic modifications. Therefore, it is advisable to closely control these conditions to minimize the risk of mosaicism and optimize the efficiency of the CRISPR/Cas9 system in larch.

In summary, this research introduces a novel application of CRISPR/Cas9 RNP complexes for targeted genome editing in larch, establishing an unprecedented method for generating mutant phenotypes within this species. It confirms the effectiveness of a DNA-free strategy in conifers, avoiding the complications associated with traditional transgenic methods while promoting sustainable forestry by maintaining genomic integrity. This innovative non-transgenic gene editing approach in larch holds considerable potential for minimizing regulatory obstacles, accelerating breeding cycles, and enhancing the integration of gene-edited trees into commercial forestry practices. This breakthrough represents a crucial advancement in the sustainable genetic enhancement of forestry species, paving the way for broad implementation and possible commercialization of genome-edited conifer plants.

## Conclusions

In this study, the optimization of parameters for the particle bombardment transformation of embryogenic callus were explored and the editing efficiency of the maker gene targets verified *in vitro*. A biolistic delivery system was employed to introduce the CRISPR/Cas9 RNP complexes into embryogenic calluses, successfully achieving edited embryogenic calluses and mutants. The present results highlight the feasibility of using CRISPR/Cas9 genome editing in the coniferous tree species, larch, through the direct delivery of RNPs. The advantages and disadvantages of this method were systematically analyzed, providing a comprehensive evaluation of its practical utility. Among the advantages is the DNA-free nature of this genome editing approach, which is particularly crucial in avoiding the integration of foreign DNA into the host genome—a significant concern in plant genetic manipulation. This method allows for precise and clean editing, minimizing regulatory hurdles associated with genetically modified organisms and facilitating the development of mutant plants that are more likely to be accepted in various markets. Another notable benefit is the possibility of applying this technique to large and complex genomes, such as those of coniferous trees. The success of this approach in larch suggests that it can be extended to other species with similarly complex genomes, potentially revolutionizing forest biotechnology by enabling the creation of genetically enhanced trees with desirable traits such as disease resistance, improved wood quality, or enhanced growth rates.

## SUPPLEMENTARY DATA

Supplementary data to this article can be found online.

## Data Availability

All data generated or analyzed during this study are included in this published article and its supplementary information files.
