# Supplementary material for: CRISPR/Cas9 ribonucleoprotein mediated DNA-free genome editing in larch
Source: For Res (Fayettev). 2024 Oct 31;4:e036. doi: 10.48130/forres-0024-0033 (PMC11564729; doi:10.48130/forres-0024-0033)
Supplement: Supplementary file 1 — Supplementary data to this article can be found online. [file FR-2024-4-0033-S1.zip › 10.48130_forres-0024-0033-Suppl-FigureS1.pdf]

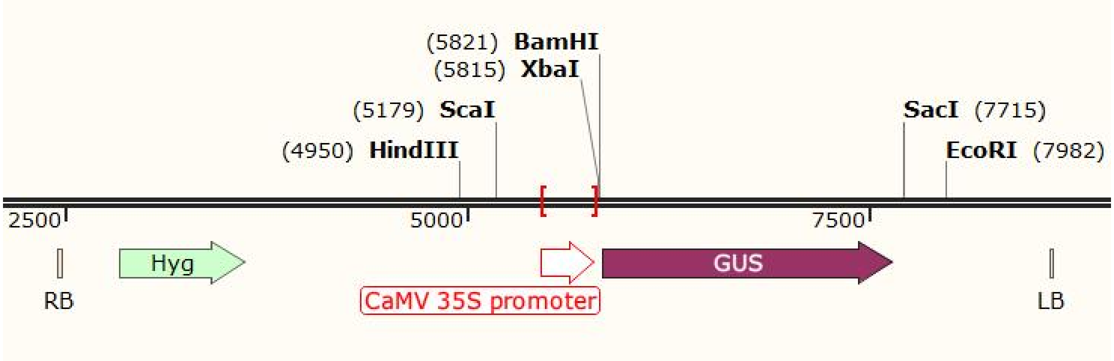

Figure S1. Schematic representation of T-DNA region in *pBI121-GUS* plasmid. RB, right border; LB, left border; Hyg, hygromycin resistance; CaMV 35S promoter, cauliflower mosaic virus 35S promoter; GUS, coding region of the GUS gene. Hind III=unique Hind III restriction site within T-DNA; Sac I=two Sac I restriction site within T-DNA; *EcoR I*=unique *EcoR I* restriction site within T-DNA; *BamH I*=unique *BamH I* restriction site within T-DNA.
