# Supplementary material for: CRISPR/Cas9 ribonucleoprotein mediated DNA-free genome editing in larch
Source: For Res (Fayettev). 2024 Oct 31;4:e036. doi: 10.48130/forres-0024-0033 (PMC11564729; doi:10.48130/forres-0024-0033)
Supplement: Supplementary file 1 — Supplementary data to this article can be found online. [file FR-2024-4-0033-S1.zip › 10.48130_forres-0024-0033-Suppl-FigureS3.pdf]

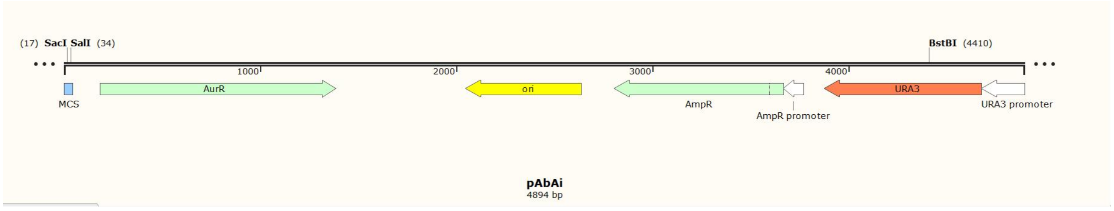

Figure S**3.** Schematic illustration of the full-length plasmid pAbAi. MCS, multiple cloning site; AurR, aureobasidin A-resistant; AmpR, ampicillin resistance; Sac I=unique Sac I restriction site within the plasmid; *Sal I*=unique *Sal I* restriction site within the plasmid; *BstB I*=unique *BstB I* restriction site within the plasmid
