# Supplementary material for: CRISPR/Cas9 ribonucleoprotein mediated DNA-free genome editing in larch
Source: For Res (Fayettev). 2024 Oct 31;4:e036. doi: 10.48130/forres-0024-0033 (PMC11564729; doi:10.48130/forres-0024-0033)
Supplement: Supplementary file 1 — Supplementary data to this article can be found online. [file FR-2024-4-0033-S1.zip › 10.48130_forres-0024-0033-Suppl-FigureS4.pdf]

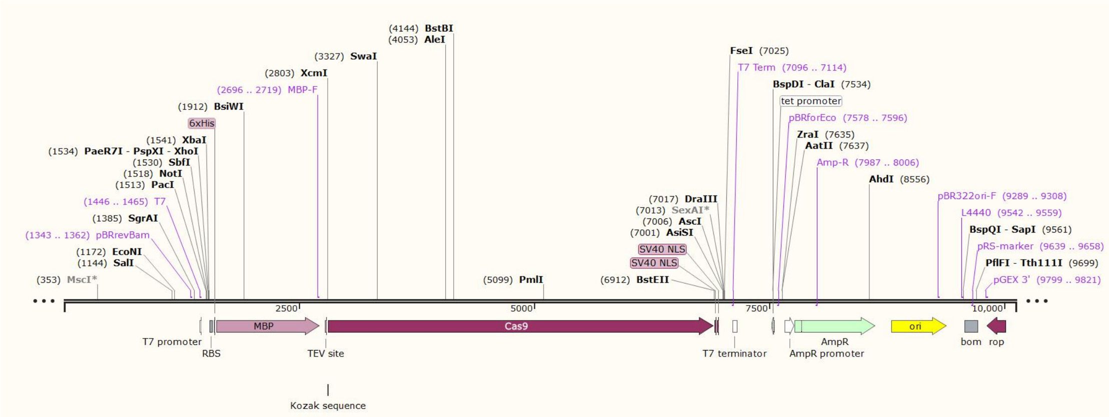


**Figure** S**4.** Representation of the full-length plasmid PMJ915. Streptococcus pyogenes Cas9 with two C-terminal SV40 NLS for nuclear localization; MBP, maltose binding protein labels; AmpR, ampicillin resistance; Multiple cloning sites for efficient gene insertion.
