# Supplementary material for: CRISPR/Cas9 ribonucleoprotein mediated DNA-free genome editing in larch
Source: For Res (Fayettev). 2024 Oct 31;4:e036. doi: 10.48130/forres-0024-0033 (PMC11564729; doi:10.48130/forres-0024-0033)
Supplement: Supplementary file 1 — Supplementary data to this article can be found online. [file FR-2024-4-0033-S1.zip › 10.48130_forres-0024-0033-Suppl-TableS1.pdf]

Table S1. Types of culture media used in the study.

Medium name	Function	Basal medium	Plant Growth Regulators
BM1	Proliferation of embryogenic callus	BM	0.2 μM 6-BA, 0.4 μM 2,4-D, 0.2 μM KT
BM2	Transition before maturation	BM	none
BM3	Maturation of somatic embryos	BM	30 mg/L ABA
BM4	Hygromycin resistant callus selection	BM	2 μM 6-BA, 4 μM 2,4-D, 2 μM KT
1/2MS	Germination of somatic embryos	MS	none
