# Supplementary material for: CRISPR/Cas9 ribonucleoprotein mediated DNA-free genome editing in larch
Source: For Res (Fayettev). 2024 Oct 31;4:e036. doi: 10.48130/forres-0024-0033 (PMC11564729; doi:10.48130/forres-0024-0033)
Supplement: Supplementary file 1 — Supplementary data to this article can be found online. [file FR-2024-4-0033-S1.zip › 10.48130_forres-0024-0033-Suppl-TableS2.pdf]

Table S2. Primers used for *GUS* gene analyses.

Description	Primer name	Sequence (5'-3')
PCR analysis	GUS-F1	ATGTTACGTCCTGTAGAAACCCC
	GUS-R1	CCTTCACCCGGTTGCCAGAGGTG
RT-PCR analyses	GUS-F2	CCGTGGTGACGCATGTCGCGC
	GUS-R2	ATGCCGACGCGAAGCGGGTAG
Internal reference actin	GAPDH-F	ATTGGAAGACTCGTCGCT
	GAPDH-R	ACCGAAAACAGCCACAGG
