# Supplementary material for: CRISPR/Cas9 ribonucleoprotein mediated DNA-free genome editing in larch
Source: For Res (Fayettev). 2024 Oct 31;4:e036. doi: 10.48130/forres-0024-0033 (PMC11564729; doi:10.48130/forres-0024-0033)
Supplement: Supplementary file 1 — Supplementary data to this article can be found online. [file FR-2024-4-0033-S1.zip › 10.48130_forres-0024-0033-Suppl-TableS3.pdf]

Table S3. Primers used for *LkPDS* gene analysis.

Description	Primer name	Sequence (5'-3')
Cloning	PDS-F	ATGCAAGGCCTTCTTTGCT
	PDS-R	CTATGCAAAGGCTGCTTCA
Fragment sequence	SacI-PF	atcgGAGCTCATGCAAGGCCTTCTTTGCT
	SalI-PR	atcgTCCCCATCATCATCCTTCCATGCAGC
Detection	Detect-F	GAAAACTGAAAAACACATATG
	Detect-R	CTCTCTGATAGAAACCATCTCG
