# Supplementary material for: CRISPR/Cas9 ribonucleoprotein mediated DNA-free genome editing in larch
Source: For Res (Fayettev). 2024 Oct 31;4:e036. doi: 10.48130/forres-0024-0033 (PMC11564729; doi:10.48130/forres-0024-0033)
Supplement: Supplementary file 1 — Supplementary data to this article can be found online. [file FR-2024-4-0033-S1.zip › 10.48130_forres-0024-0033-Suppl-TableS5.pdf]

Table S5. Details of primer design and gRNA synthesis for the study.

Primer species and their final concentrations

Name	Final concentration
gRNA_primer	500 nM
T25-long	10 μM
BS6	500 nM
BS7	500 nM

PCR reaction to produce double stranded DNA template

Reagents	Volume
gRNA_primer	2 μl
BS6	2 μl
T25-long	5 μl
BS7	5 μl
dNTP Mix(10mM)	1 μl
10*PCR Buffer	5 μl
rTaq	1 μl
Nuclease free Water	29 μl

PCR Setting

Step	Temperature (°C)	Time (min)
Step 1 Denature	95 °C	1 min
Step 2 Denature	95 °C	10 s
Step 3 Anneal	59 °C	10 s
Step 4 Elongation	72 °C	10 s
	From step 2- step 4 40 cycles	
Step 5 Elongation	72 °C	30 s
Preservation	4 °C	Forever
