# Supplementary material for: CRISPR/Cas9 ribonucleoprotein mediated DNA-free genome editing in larch
Source: For Res (Fayettev). 2024 Oct 31;4:e036. doi: 10.48130/forres-0024-0033 (PMC11564729; doi:10.48130/forres-0024-0033)
Supplement: Supplementary file 1 — Supplementary data to this article can be found online. [file FR-2024-4-0033-S1.zip › 10.48130_forres-0024-0033-Suppl-TableS6.pdf]

Table S6. Instructions for preparing target DNA and Cas9 recombinant protein.

gRNA in vitro transcription (NEB T7 quick high yield RNA synthesis kit)

Reagents	Volume
NTP Buffer Mix	7.5 µl
Template DNA	8 µl
T7 RNA Polymerase	1.5 µl
Nuclease free water	3 µl
Total reaction volume	20µl
Incubate reactions in a PCR machine at 37 °C for 4 h.	

The linearized system of recombinant vector

Reagents	Volume
DNA sample	20 µL
Restriction enzyme	10 µL
10 × Buffer	20 µL
dH2O	150 µL
Incubate at 37 °C for 3 h	

In vitro cleavage assay

Reagent	Volume
Cas9 recombinant protein (1mg/mL)	2 µL
gRNA	2 µL
Linearized target DNA	12 µL
Phosphate buffer (pH 7.5, 1 M)	0.8 µL (50 mM final)
MgCl2 (100 mM)	1 µL (5 mM final)
DTT (1 M)	0.02 µL (1 mM final)
PBS	2.18 µL
Total volume	20 µL
Incubate assays at 37 °C for 1 h	
