# Supplementary material for: CRISPR/Cas9 ribonucleoprotein mediated DNA-free genome editing in larch
Source: For Res (Fayettev). 2024 Oct 31;4:e036. doi: 10.48130/forres-0024-0033 (PMC11564729; doi:10.48130/forres-0024-0033)
Supplement: Supplementary file 1 — Supplementary data to this article can be found online. [file FR-2024-4-0033-S1.zip › 10.48130_forres-0024-0033-Suppl-TableS7.pdf]

Table S7. Primer sequences for amplifying genomic fragments covering the target sites.

	Forward primer(5'-3')	Reverse primer(5'-3')
Target 1	ATGCAAGGCCTTCTTTGCTTTTC	CACAGAGCGCATAGCAGAAACT
Target 2	GCAGTCTGTCATCTGCGAGGAG	AGCAGATAAAGCAGCAGCTTCC
